# Muscle Free Fatty-Acid Uptake Associates to Mechanical Efficiency During Exercise in Humans

**DOI:** 10.3389/fphys.2018.01171

**Published:** 2018-08-21

**Authors:** Marko S. Laaksonen, Heikki Kyröläinen, Jukka Kemppainen, Juhani Knuuti, Kari K. Kalliokoski

**Affiliations:** ^1^Swedish Winter Sports Research Centre, Department of Health Sciences, Mid Sweden University, Östersund, Sweden; ^2^Neuromuscular Research Centre, Biology of Physical Activity, Faculty of Sport and Health Sciences, University of Jyväskylä, Jyväskylä, Finland; ^3^Turku PET Centre, University of Turku, Turku, Finland; ^4^Department of Clinical Physiology and Nuclear Medicine, University of Turku, Turku, Finland

**Keywords:** economy, free fatty-acid, metabolism, skeletal muscle, oxygen uptake

## Abstract

Intrinsic factors related to muscle metabolism may explain the differences in mechanical efficiency (ME) during exercise. Therefore, this study aimed to investigate the relationship between muscle metabolism and ME. Totally 17 healthy recreationally active male participants were recruited and divided into efficient (EF; *n* = 8) and inefficient (IE; *n* = 9) groups, which were matched for age (mean ± SD 24 ± 2 vs. 23 ± 2 years), BMI (23 ± 1 vs. 23 ± 2 kg m^−2^), physical activity levels (3.4 ± 1.0 vs. 4.1 ± 1.0 sessions/week), and $\dot{V}$O_2_peak (53 ± 3 vs. 52 ± 3 mL kg^−1^ min^−1^), respectively, but differed for ME at 45% of $\dot{V}$O_2_peak intensity during submaximal bicycle ergometer test (EF 20.5 ± 3.5 vs. IE 15.4 ± 0.8%, *P* < 0.001). Using positron emission tomography, muscle blood flow (BF) and uptakes of oxygen (m$\dot{V}$O_2_), fatty acids (FAU) and glucose (GU) were measured during dynamic submaximal knee-extension exercise. Workload-normalized BF (EF 35 ± 14 vs. IE 34 ± 11 mL 100 g^−1^ min^−1^, *P* = 0.896), m$\dot{V}$O_2_ (EF 4.1 ± 1.2 vs. IE 3.9 ± 1.2 mL 100 g^−1^ min^−1^, *P* = 0.808), and GU (EF 3.1 ± 1.8 vs. IE 2.6 ± 2.3 μmol 100 g^−1^ min^−1^, *P* = 0.641) as well as the delivery of oxygen, glucose, and FAU, as well as respiratory quotient were not different between the groups. However, FAU was significantly higher in EF than IE (3.1 ± 1.7 vs. 1.7 ± 0.6 μmol 100 g^−1^ min^−1^, *P* = 0.047) and it also correlated with ME (*r* = 0.56, *P* = 0.024) in the entire study group. EF group also demonstrated higher use of plasma FAU than IE, but no differences in use of plasma glucose and intramuscular energy sources were observed between the groups. These findings suggest that the effective use of plasma FAU is an important determinant of ME during exercise.

## Introduction

Maximal oxygen uptake ($\dot{V}$O_2_max), $\dot{V}$O_2_ at lactate threshold, and work economy are the three main factors determining endurance performance (Bassett and Howley, 2000). In which extend these variables contribute to performance is not totally clear and the contribution also depends on performance level of study populations as summarized recently (Borgen, 2018). A proper technique is believed to associate with higher efficiency, also in cycling which is in technical terms relatively simple exercise type (Leirdal and Ettema, 2011).

The gross mechanical efficiency (ME) reflecting the work economy is defined as a ratio of the mechanical work to the metabolic energy expended (Stainbsy et al., 1980; Cavanagh and Kram, 1985), and it is usually higher in endurance-trained compared to moderately trained individuals (Hopker et al., 2007). That makes it as an important factor in endurance performance (Joyner, 1991; Coyle, 1999; Joyner and Coyle, 2008). ME in endurance trained cyclists usually varies from 18 to 28% (Coyle et al., 1992; Lucia et al., 2002) and some evidence suggest that ME can be improved, within those limits, by 1–3% per year with continued endurance training (Joyner and Coyle, 2008; Hopker et al., 2009).

Muscle metabolism is one of the key factors contributing to ME (Perrault, 2006). It is well known that endurance training shifts muscle energy metabolism from carbohydrates toward increased fat oxidation during the same relative low to moderate exercise intensities (Coggan et al., 1990; Turcotte et al., 1992; Bergman et al., 1999). It has also been shown that in trained participants, plasma fatty acids (FAU) are the major source of energy during prolonged exercise, e.g., at moderate exercise intensities such as cycling at 65% of $\dot{V}$O_2_max intensity (Romijn et al., 1993). Moreover, FAU are almost the sole source of energy during low exercise intensities (Romijn et al., 1993). On the other hand, contribution of blood glucose and muscle glycogen stores increase and they are the main source of energy at high exercise intensities (Romijn et al., 1993). Thus, the shift in relative contributions of fats and carbohydrates may explain the changes in ME. Body fat content may play an important role in this as seen in obese participants (Jabbour and Iancu, 2015). However, improved fat oxidation by endurance training has been explained by enhanced FAU delivery (Saltin et al., 1983) that is due to increased capillary density (Andersen and Henriksson, 1977) although it is has been recently linked more to transport mechanisms from blood to mitochondria likely diminishing the role of mitochondrial content in fuel selection (Yoshida et al., 2013). However, increased capillary density is one of the factors explaining increased muscle blood flow (BF) capacity after endurance training (Armstrong and Laughlin, 1984; Saltin, 1985; Kalliokoski et al., 2001). Thus, changes in ME may also be related to changes in muscle BF.

Taken together, the shift in relative contributions of fats and carbohydrates may explain the changes in ME. Therefore, the present study aimed to investigate whether muscle BF and uptake of glucose (GU) and FAU at rest and during standardized exercise are different in the groups of participants with different ME during a ramp bicycle exercise test. Muscle oxygen uptake (m$\dot{V}$O_2_) was also measured and used to calculate the total energy consumption and the use of intramuscular energy sources. As muscle metabolism is also partly regulated by hormones such as catecholamines and insulin, the concentrations of these hormones were determined to complete the picture. Thus, based on the findings related to endurance training and ME, the hypothesis was that more efficient participants have increased FAU and decreased GU in their working muscles.

## Materials and Methods

### Participants

Totally 17 healthy physically active male participants without any specific background in endurance training were recruited into the study. None of them were characterized as endurance athletes but they were engaged in leisure-time physical activity in average 3.8 ± 1.0 times and 220 ± 80 min per week. They gave their written informed consent to participate to the study after being informed of the purpose, nature, and potential risks involved prior to initiation of the experiments. The participants were instructed to avoid all exercise for 2 days, restrict their intake of caffeinated beverages for 24 h and fast overnight for 10 h prior to each test occasion. This study was carried out in accordance with the recommendations of Ethical Committee of the Hospital District of South-Western Finland. The protocol was approved by the Ethical Committee of the Hospital District of South-Western Finland and all participants gave written informed consent in accordance with the Declaration of Helsinki.

### Procedure and Study Design

**FIGURE 1:**
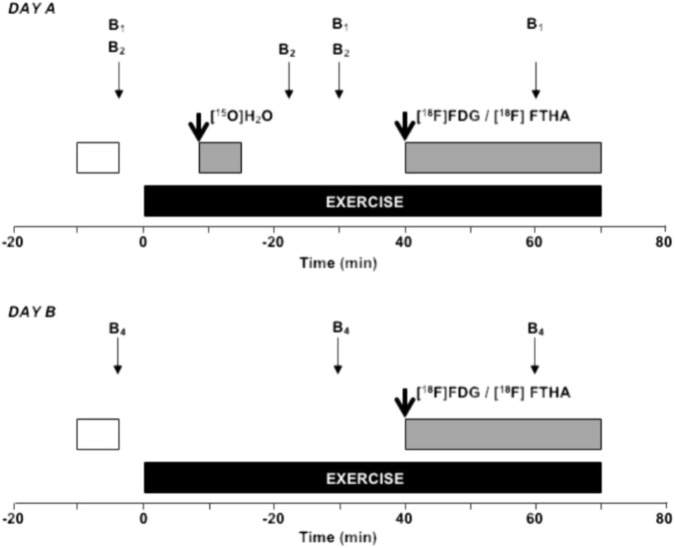
Study design during the PET experiments. During the day A, the study subject was carefully attached to the ergometer and PET-scanner which was followed by of 20 min of rest. Ten minutes before the start of exercise a transmission scan (*white box*) of femoral region was performed. Thereafter, arterial (a. *radialis*) and venous (v. *femoralis*) blood samples (*thin arrows*) for analysis of plasma glucose, free fatty acids, and lactate (B_1_) as well as for blood O_2_ content (B2) were taken at rest and during exercise. In addition, venous concentrations (*antecubital* vein) for insulin, epinephrine, and norepinephrine (B_1_) were taken at rest and during exercise. After 8 min of dynamic knee-extension exercise the muscle BF was measured using PET-scanning (*gray box*) after injection (*thick arrow*) of [^15^O]H_2_O. In addition, muscle oxygen uptake was determined using BF data and arterio-venous blood samples for O_2_ content at 23 and 30 min of exercise (B_2_). Finally, after 40 min of exercise muscle glucose or fatty acid uptake was measured during 30 min period using PET-scanning (gray box) after injection of [^18^F]FDG or [^18^F]FTHA, respectively. During the day B, similar exercise protocol was used but only muscle fatty acid or glucose uptake was measured with PET using arterialized blood samples for plasma fatty acid or glucose (B_4_), respectively. The order of day A and B was randomized between participants.

One to 2 weeks before the positron emission tomography (PET) experiments all participants performed a maximal and submaximal bicycle ergometer test in order to determine the peak oxygen uptake ($\dot{V}$O_2_peak) and peak power output (PPO) as well as submaximal oxygen consumption (O_2_ cost) and ME. Submaximal O_2_ cost and ME were measured and calculated, respectively, at 45% of $\dot{V}$O_2_peak exercise intensity to correspond most closely the exercise intensity during one-legged knee-extension exercise during PET scanning due to the fact that PET measurement does not allow significant body motion in the scanner and therefore, only low exercise intensity during cycling exercise was used. Thereafter, the participants were allocated into the EF (*n* = 8) and inefficient (IE; *n* = 9) groups based on their ME (EF 20.5 ± 1.3% vs. IE 15.4 ± 0.3%, *P* < 0.01) with similar $\dot{V}$O_2_peak, PPO, age, physical activity level, and body mass index (**Table 1**). The PET experiment was then performed during two consecutive days (*A* and *B*) which were randomized between the participants (**Figure 1**). During the day *A* three catheters were inserted: One into an antecubital vein for injection of tracer, the second into the opposite radial artery and the third into the femoral vein for blood sampling. Thereafter, participants performed a steady-rate dynamic one-legged knee-extension exercise for 70 min during which the muscle BF, m$\dot{V}$O_2_ and GU or FAU were measured together with a measurement of knee-extensors’ force production as well as blood sampling. During the day *B*, one catheter was inserted into antecubital vein for injection of tracer and another one into the opposite antecubital vein for blood sampling. After this, similar exercise bout as during day *A* was performed and muscle FAU or GU was measured together with blood sampling.

**Table 1:** Physical activity levels as well as anthropometric and hematological characteristics of study participants in the efficient (EF) and inefficient (IE) groups.

	EF (*n* = 8)	IE (*n* = 9)
Physical activity (sessions/week)	3.43 ± 0.98	4.13 ± 0.99
Physical activity (min/session)	64.29 ± 11.34	52.50 ± 26.59
Age (years)	23.88 ± 2.03	23.22 ± 2.11
Body mass (kg)	74.61 ± 9.22	75.73 ± 6.39
Body height (m)	1.79 ± 0.06	1.81 ± 0.05
Body mass index (kg m^−2^)	23.14 ± 1.42	23.20 ± 1.64
Body surface are (m^2^)	1.93 ± 0.15	1.95 ± 0.10
Body fat (%)	11.31 ± 4.99	12.36 ± 4.67
Erythrocytes (E12 L^−1^)	4.77 ± 0.18	4.67 ± 0.21
Hemoglobin (g L^−1^)	145.75 ± 3.73	143.33 ± 5.94
Hematocrit (%)	40.50 ± 1.60	40.67 ± 1.32
MCV (fL)	85.63 ± 2.62	87.89 ± 3.14
MCH (pg cell^−1^)	30.75 ± 1.16	30.89 ± 1.36
Leukocytes (E9 L^−1^)	4.75 ± 0.71	5.19 ± 0.83
Platelets (E9 L^−1^)	205.25 ± 41.95	205.22 ± 41.30
Glucose (mmol L^−1^)	5.62 ± 0.40	5.56 ± 0.47
Plasma fatty acids (mmol L^−1^)	0.46 ± 0.28	0.35 ± 0.13
Lactate (mmol L^−1^)	0.94 ± 0.33	0.89 ± 0.25

*Values are mean ±SD.*

### Exercise During Bicycle Test and PET-Experiments

For determining the $\dot{V}$O_2_peak and PPO an incremental bicycle ergometer (Tunturi E980, Turku, Finland) test was performed starting at 75 W with 25 W increases every 2 min until exhaustion. After 30 min of recovery a submaximal bicycle exercise for 4 min was performed at 45% of $\dot{V}$O_2_peak in order to determine the ME.

The exercise protocol during PET experiments consisted of dynamic, one-legged (right leg) knee-extension exercise for 70 min (both days *A* and *B*) at workload 38 ± 12 N using a specially manufactured dynamometer. The individual resistance was based on the maximal isometric knee-extension force. This relatively low exercise intensity was chosen in order (i) to ensure that all study participants managed to perform the entire exercise protocol based on pilot experiment and (ii) to diminish the possible motion artifacts during PET-scanning (Laaksonen et al., 2003). Force production during knee-extension exercise was, therefore, measured with a force transducer located anterior to the tibia and above the malleolus medialis (Laaksonen et al., 2003). Contraction frequency was self-paced based on an earlier report showing that the freely chosen frequency is the most EF (Cavanagh and Williams, 1982).

### Production of PET Tracers

For production of ^15^O (*t*_1/2_ = 123 s), a low-energy deuteron accelerator, Cyclone 3, was used (Ion Beam Application, Loucain-la-Neuve, Belgium). [^15^O]O_2_ was produced by the nuclear reaction [^14^N](d,n)[^15^O] and thereafter, the positron emitting radiowater ([^15^O]H_2_O) was produced by continuously working water module as previously described (Sipilä et al., 2001). [^18^F] (*t*_1/2_ = 109 min) was produced by irradiating ^18^O-enriched water using isochronous cyclotron. [^18^F]FDG was then synthesized with an automatic apparatus described by Hamacher et al. (1986). [^18^F]FTHA was synthesized by nucleophilic radiofluorination of benzyl 14(R,S)-tosuloxy-6-thia-heptadecanoate as previously described (Maki et al., 1997). [^15^O]H_2_O, [^18^F]FDG, and [^18^F]FTHA were used for measurements of muscle BF, GU, and FAU, respectively.

### Image Acquisition and Processing

An 8-ring ECAT 931/08 tomograph (Siemens/CTI, Knoxville, TN, United States) was used for 2D image acquisition. The axial resolution was 6.7 mm and in-plane resolution 6.5 mm. To correct for photon attenuation, a transmission scan with removable ring source containing ^68^Ge for 5 min was performed. For measurement of muscle BF, [^15^O]H_2_O was injected intravenously with an automatic injection system (Sipilä et al., 2001) and dynamic scanning started for 6 min. To determine the input function for BF, arterial blood was continuously withdrawn with a pump at a speed of 6 mL min^−1^ from the radial artery, and the radioactivity concentration was measured using a 2-channel online detector system (Scanditronix, Uppsala, Sweden), cross-calibrated with an automatic gamma counter (Wizard 1480 3″, Wallac, Turku, Finland) and PET scanner (Ruotsalainen et al., 1997). For measurement of muscle GU, [^18^F]FDG was injected intravenously as a continuous infusion with a anesthesia pump (Graseby 3400, Graseby Medical Ltd., Watford, United Kingdom) over 90 s with a simultaneous start of dynamic scan for 30 min. The blood samples for measurement of plasma radioactivity concentration were withdrawn as previously described (Nuutila et al., 1992). For determination of muscle FAU, [^18^F]FTHA was injected intravenously over 10 min. The 30 min PET scan started simultaneously with the injection. The blood samples for measurement of plasma radioactivity concentration were withdrawn every 2 min during the first 20 min, and thereafter every 5 min. The non-metabolized fraction of [^18^F]FTHA was determined using high-performance liquid chromatography from eight blood samples (0, 2, 5, 10, 15, 20, 25, and 30 min from the beginning of the FTHA injection). All PET data were corrected for dead time, decay, and measured photon attenuation. PET images were processed using the ordered subsets expectation maximization and median root prior reconstruction algorithm (2D OSEM-MRP) with 150 iterations and the Bayesian coefficient 0.3 (Alenius and Ruotsalainen, 1997).

Muscle BF was calculated using an autoradiographic method as mentioned earlier (Ruotsalainen et al., 1997; Laaksonen et al., 2003). To calculate muscle GU and FAU, first fractional rate of tracer ([^18^F]FDG and [^18^F]FTHA) uptake was calculated using graphical analysis (Patlak and Blasberg, 1985) from tissue and plasma time activity curves. The rate of muscle GU and FAU was then calculated by multiplying the fractional tracer uptake by the plasma glucose or plasma FAU concentration, respectively. As the contraction frequency was freely chosen by the participants, the muscle BF, GU, FAU, and m$\dot{V}$O_2_ values obtained from the exercising leg were indexed to the same workload by multiplying the individual value of these variables with the ratio between the average rotational work of all participants and the individual rotational work.

### Region of Interests

The region of interest was drawn on the both quadriceps femoris muscles (QF; exercising right leg and resting left leg) in four consecutive cross-sectional planes, carefully avoiding large blood vessels.

### Whole Body Oxygen Consumption and Mechanical Efficiency

Oxygen consumption ($\dot{V}$O_2_) during bicycle exercise was measured using Cosmed K4b^2^ (Cosmed, Rome, Italy) with breath-by-breath sampling method. Data was averaged thereafter for each 30 s time period. $\dot{V}$O_2_peak was determined as the highest averaged 30 s $\dot{V}$O_2_ value during the incremental bicycle test. ME was determined as a ratio of mechanical work (workload [W] × 0.014) and metabolic energy expended using $\dot{V}$O_2_ and respiratory exchange ratio during submaximal (45% of $\dot{V}$O_2_peak) bicycle exercise × 100%.

### Biochemical Analysis

During bicycle test, capillary blood samples for determination of blood lactate concentration were obtained from a fingertip at rest, at the end of submaximal workload and 3 min after the cessation of maximal bicycle test. These samples were further analyzed using an enzymatic method (Biochemica Boehringer, Mannheim, Germany). During the day *A* of PET experiments, venous blood samples from the antecubital vein were withdrawn at rest and after 30 and 60 min of exercise in order to determine the concentrations of blood lactate as well as plasma glucose or plasma FAU. Similarly, blood samples for analyzing the concentration of catecholamines (epinephrine and norepinephrine) and insulin were taken at rest and after 30 and 60 min of exercise (**Figure 1**). In addition, both arterial (a. *radialis*) and venous (v. *femoralis*) blood samples were taken at rest and after 23 and 30 min of exercise for determination of blood CO_2_ and O_2_ content. During the day *B*, arterialized blood samples were taken from antecubital vein at rest and after 30 and 60 min of exercise in order to measure plasma FAU or glucose concentrations depending on if muscle FAU or GU was measured, respectively. Plasma glucose concentration was measured with Analox Glucose Analyser GM9 (Analox Instruments Limited, London, United Kingdom). Modular P800 automatic analyzer (Roche Diagnostics GmbH, Mannheim, Germany) was used to analyze plasma FAU and blood lactate (B-La; coefficient of variation 6.2%). Catecholamines (coefficient of variance 15.0% for epinephrine and 16.1% for norepinephrine at 2.5 and 24.4 ng mL^−1^ levels, respectively; Hjemdahl et al., 1979) and insulin (Kuzuya et al., 1977) concentrations were analyzed as described earlier. Arterio-venous blood samples for CO_2_ and O_2_ content were analyzed by Ciba-Corning 865 (Ciba-Corning Diagnostics Corp., Medfield, MA, United States).

### Other Measurements and Calculations

The PPO was calculated using the formula by Kuipers et al. (1985):

(1) $$\text{PPO}=W_{\text{E}}+\left(\frac{t_{\text{E}}}{t}\times 25\;W\right)$$

where *W*_E_ is the power output of the last completed workload (*W*), *t*_E_ is the duration of the final workload (seconds), and *t* is the duration of each workload (seconds) (Kuipers et al., 1985). During the knee-extension exercise (PET experiment), the angular displacement for each knee extension was calculated using the known range of the knee joint angle and the length of the moment arm. Performed rotational work per 1 min was then calculated as: Torque × angular displacement per minute.

Muscle oxygen uptake as determined as arterio-venous O_2_ difference multiplied by muscle BF. Muscle O_2_ delivery was calculated by multiplying the arterial O_2_ content with the rate of muscle BF and further, muscle O_2_ extraction was determined as m$\dot{V}$O_2_ divided by muscle O_2_ delivery × 100%. Muscle respiratory quotient (RQ) was calculated as a ratio between produced CO_2_ and consumed O_2_ based on arterio-venous blood sampling.

Muscle total energy expenditure (EE_tot_) as well as the energy derived from fats (FAT_tot_) and carbohydrates (CHO_tot_) during exercise were calculated as m$\dot{V}$O_2_ × caloric equivalent based on muscle RQ. Further, energy derived from blood fatty acids (FFA_blood_) taken up by exercising muscle was calculated as 1,065.8 kcal mol^−1^ × FAU assuming a complete oxidation of FAU. Similarly, energy derived from blood glucose (GLUC_blood_) taken up by muscle was calculated as 277.4 kcal mol^−1^ × GU also assuming a complete oxidation of glucose. The amount of energy derived from intramuscular fat (FAT_intra_) and carbohydrates (CHO_intra_) were calculated as FAT_tot_ - FFA_blood_ and CHO_tot_ - GLUC_blood_, respectively. Both of these calculations were based on the assumption that all plasma FAU and glucose taken up by muscle cells are entered to substrate oxidation. Aerobic ATP production was calculated according to the method by Hinkle and Yu (1979). Glucose and FAU delivery were calculated as a product of muscle BF and arterial plasma glucose and FAU concentration, respectively. Further, extraction of glucose and FAU were calculated as the uptake divided by the delivery of respective substrates.

### Statistical Analyses

All statistical evaluations were performed with PASW Statistics 18 statistical software release 18.0.0 (SPSS Inc., Chicago, IL, United States) using the alpha level of 0.05. The normal distribution of all parameters was first tested using Shapiro–Wilks test. Two-tailed Student’s *t*-test for independent samples was utilized when comparing the groups. Changes in insulin, epinephrine and norepinephrine concentrations (time × group) were tested with two-way ANOVA. In addition, the differences in the use of FFA_blood_ and GLUC_blood_ (substrate × group) as well as FAT_intra_ and CHO_intra_ (substrate × group) were tested with two-way ANOVA. The magnitude of differences were expressed as standardized mean differences (effect size, ES; Cohen’s *d* and η^2^ for Student’s *t*-test and ANOVA, respectively) where the criteria for interpreting the magnitudes of ES were <0.2 trivial, 0.2–0.6 small, 0.6–1.2 moderate, 1.2–2.0 large, and >2.0 very large (Hopkins et al., 2009). Also the observed power was calculated for main variables. Pearson’s product-moment correlation coefficient was utilized when testing the linear relationship between parameters. Study characteristics is presented as mean ± SD. Other data is as mean (95% confidence interval, CI).

## Results

### Bicycle Exercise Test

Peak power output, $\dot{V}$O_2_peak, and maximal blood lactate concentration in the both groups are presented in **Table 2**. Performance in terms of PPO and $\dot{V}$O_2_peak was comparable between the groups. No difference in blood lactate concentration was observed between the groups at this exercise intensity (**Table 2**).

**Table 2:** Power output (PO) and physiological measures during submaximal (45% of $\dot{V}$O_2_peak) and incremental maximal bicycle exercise test in efficient (EF) and inefficient (IE) groups.

	EF (*n* = 8)	IE (*n* = 9)	Effect size	Power
PO45% (W)	121.25 (114.38, 128.12)	109.44 (100.73, 118.16)	0.15	0.47
PO45% (W kg^−1^)	1.64 (1.53, 1.74)	1.45 (1.33, 1.57)^∗^	1.08	0.54
PPO @ $\dot{V}$O_2_peak (W)	292.97 (278.53, 307.41)	268.75 (245.49, 292.01)	0.83	0.39
PPO @ $\dot{V}$O_2_peak (W kg^−1^)	3.96 (3.70, 4.22)	3.56 (3.24, 3.88)	0.92	0.42
$\dot{V}$O_2_45% (mL kg^−1^ min^−1^)	22.67 (20.71, 24.62)	26.44 (24.62, 28.25)^#^	1.32	0.65
$\dot{V}$O_2_peak (mL kg^−1^ min^−1^)	52.79 (47.99, 57.60)	51.60 (45.86, 57.35)	0.15	0.05
B-La45% (mmol L^−1^)	2.31 (1.52, 3.10)	3.02 (2.52, 3.52)	0.56	0.31
B-La_max_ (mmol L^−1^)	11.74 (10.84, 12.64)	12.17 (11.16, 13.18)	0.36	0.09
Mechanical efficiency (%)	20.54 (18.08, 23.00)	15.35 (14.82, 15.88)^§§^	2.01	0.84

*Values are means (95% CI). PO, power output; PPO, peak power output; $\dot{V}$O_2_, oxygen consumption; B-La, blood lactate. ^∗^P = 0.042, ^#^P = 0.014, and ^§§^ P < 0.001 between groups.*

### Muscle BF and m$\dot{V}$O_2_

**FIGURE 2:**
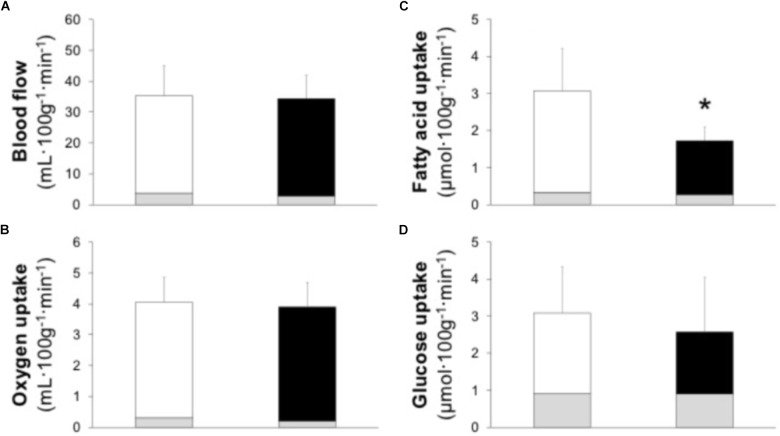
Skeletal muscle blood flow **(A)** and uptake of oxygen **(B)**, plasma fatty acids **(C)**, and glucose **(D)** in efficient (*white bars*) and inefficient (*black bars*) groups during one-legged knee-extension exercise. Gray bars present the resting values. Data is mean ± (95% CI). ^∗^*P* = 0.047 compared to efficient group.

Muscle BF [EF 3.8 (3.1, 4.6) vs. IE 2.9 (2.1, 3.7) mL 100 g^−1^ min^−1^, *P* = 0.279] and m$\dot{V}$O_2_ [EF 0.3 (0.2, 0.3) vs. IE 0.2 (0.1, 0.3) mL 100 g^−1^ min^−1^, *P* = 0.406) in resting as well as in exercising muscle (**Figures 2A,B**; BF effect size 0.07, power 0.04; m$\dot{V}$O_2_ effect size 0.13, and power 0.05) were at the same level between the groups. Also, the resting O_2_ delivery [EF 0.8 (0.6, 1.0) vs. IE 0.6 (0.4, 0.9) mL 100 g^−1^ min^−1^, *P* = 0.357) and O_2_ extraction [EF 33 (30, 37) vs. IE 35 (33, 38) %, *P* = 0.372] were at the same level between the groups. Similarly, no difference in exercising muscle O_2_ delivery, O_2_ extraction, or aerobic ATP production between the groups was observed (**Table 3**).

**Table 3:** Exercising muscle substrate delivery and extraction as well as aerobic ATP production and muscle RQ in efficient (EF) and inefficient (IE) groups during one-legged knee-extension exercise.

	EF (*n* = 8)	IE (*n* = 9)	Effect size	Power
O_2_ delivery (mL 100 g^−1^ min^−1^)	7.4 (5.4, 9.4)	7.1 (5.6, 8.7)	0.09	0.04
O_2_ extraction (%)	60 (42, 79)	60 (45, 74)	0.02	0.03
Fatty acid delivery (μmol 100 g^−1^ min^−1^)	0.15 (0.09, 0.20)	0.13 (0.09, 0.16)	0.30	0.10
Fatty acid extraction (%)	25 (19, 33)	15 (10, 19)^∗^	1.20	0.58
Glucose delivery (μmol 100 g^−1^ min^−1^)	0.20 (0.15, 0.25)	0.20 (0.15, 0.26)	0.05	0.03
Glucose extraction (%)	18 (11, 24)	20 (1, 38)	0.12	0.04
Aerobic ATP production (mmol min^1^)	18 (15, 22)	18 (14, 21)	0.13	0.05
Muscle RQ	0.83 (0.78, 0.88)	0.82 (0.78, 0.86)	0.07	0.04

*Values are means (95% CI). O_2_, oxygen; RQ, respiratory quotient. ^∗^P = 0.045 between groups.*

### Muscle FAU and GU

Resting muscle FAU [EF 0.34 (0.27, 0.41) vs. IE 0.28 (0.22, 0.34) μmol 100 g^−1^ min^−1^, *P* = 0.225) and GU [EF 0.9 (0.8, 1.0) vs. IE 0.9 (0.7, 1.1) μmol 100 g^−1^ min^−1^, *P* = 0.983) were at the same level in the both groups. During exercise the delivery of both FAU and glucose was similar between the groups (**Table 3**). Muscle FAU was higher in the EF than IE group (**Figure 2C**; effect size 1.08 and power 0.52) whereas no difference was observed in muscle GU (**Figure 2D**; effect size 0.25 and power 0.08). Similarly, the EF group demonstrated higher FAU extraction during exercise whereas glucose extraction and muscle RQ during exercise were similar between the groups (**Table 3**).

### Energy Metabolism

**FIGURE 3:**
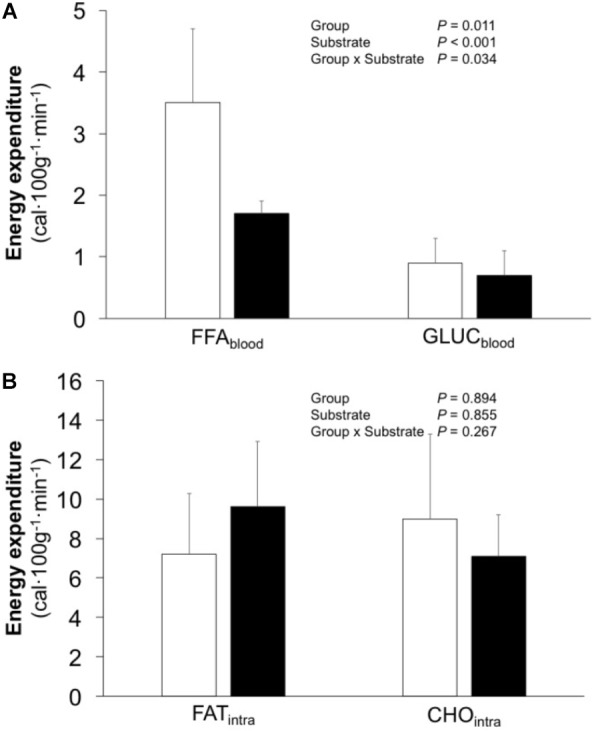
Caloric contribution from plasma fatty acids (FFA_blood_) and glucose (GLUC_blood_) **(A)** as well as from intramuscular triglycerides (FAT_intra_) and glycogen (CHO_intra_) **(B)** in efficient (*white bars*) and inefficient (*black bars*) groups during one-legged knee-extension exercise. Data is mean ± (95% CI). *P*-values refer to analysis of variance.

EE_tot_ averaged 19.6 (15.6, 23.6) and 18.8 (15.0, 22.6) cal 100 g^−1^ min^−1^ (*P* = 0.774) for EF and IE, respectively. Similarly, no difference in FAT_tot_ [EF 10.7 (7.1, 14.3) vs. IE 11.3 (7.8, 22.6) cal 100 g^−1^ min^−1^; *P* = 0.542) or GLUC_tot_ [EF 9.9 (5.7, 14.1) vs. IE 7.5 (5.4, 9.7) cal 100 g^−1^ min^−1^; *P* = 0.376) was observed. However, the EF group demonstrated higher caloric contribution from plasma FAU and glucose (effect size 1.16 and power 0.59), whereas no differences were observed for intramuscular fat and carbohydrates (**Figure 3**; effect size 0.22 and power 0.11).

### Blood Variables

Hematological characteristics were comparable between the groups (**Table 1**). During exercise, insulin concentration was similar as at rest but lower in the EF than IE group (group *P* = 0.044) (**Table 4**). Norepinephrine concentration tended to increase during exercise (time *P* = 0.055) but was similar in the both groups. Epinephrine concentration was stable during the entire experiment with no difference between the groups.

**Table 4:** Insulin, norepinephrine, and epinephrine concentrations at rest and after 30 and 60 min of exercise during one-legged knee-extension exercise in efficient (EF) and inefficient (IE) groups.

		Rest	30 min	60 min	Results (*p*-values)		
					Time	Group	Time × group
Insulin (mU L^−1^)	EF	8.2 (5.5, 13.6)	8.5 (6.8, 15.3)	6.2 (5.6, 11.8)	0.293	0.044	0.910
	IE	9.8 (8.6, 11.0)	11.0 (7.7, 14.3)	9.0 (6.9, 11.1)			
Noradrenalin (mU L^−1^)	EF	1.3 (1.1, 2.4)	1.5 (1.3, 2.8)	1.6 (1.2, 2.8)	0.055	0.667	0.808
	IE	1.3 (1.1, 1.5)	1.5 (1.3, 1.8)	1.8 (1.5, 2.0)			
Adrenalin (mU L^−1^)	EF	0.5 (0.3, 0.8)	0.5 (0.4, 0.8)	0.6 (0.4, 1.0)	0.247	0.990	0.763
	IE	0.5 (0.4, 0.6)	0.5 (0.4, 0.5)	0.6 (0.4, 0.8)			

*Values are means (95% CI).*

### Associations

**FIGURE 4:**
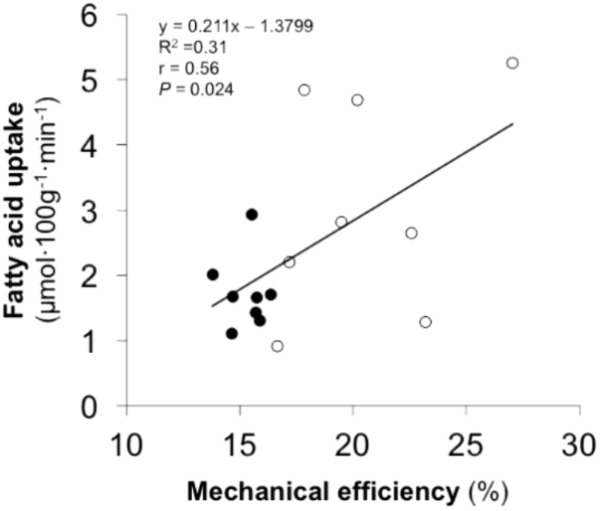
Bivariate correlation between skeletal muscle fatty acid uptake during one-legged knee-extension exercise and mechanical efficiency during cycling in the entire study population (*white circles* represents efficient participants and *black circles* represents inefficient participants).

A significant correlation was observed between ME and muscle FAU (**Figure 4**) as well as between ME and FAU extraction (*r* = 0.56, *P* = 0.020) during exercise. None of the other main variables correlated with ME or PPO.

## Discussion

In this study, it was hypothesized that ME would be associated with changes in muscle metabolism so that more EF participants would have increased muscle FAU but decreased GU. The results partly supported this hypothesis as muscle FAU was higher in more EF participants and it also correlated significantly with ME. In addition, based on direct measurements of m$\dot{V}$O_2_ and calculation of total muscle energy expenditure, no significant differences were observed between the groups in use of intramuscular energy sources or of blood glucose, but the use of plasma FAU was higher in more efficient participants.

Work economy or efficiency is one of the main components of endurance performance (Bassett and Howley, 2000; Joyner and Coyle, 2008). As efficiency is related to endurance performance and training (Paavolainen et al., 1999; Millet et al., 2002; Jones, 2006), the present study aimed to investigate the relationship between muscle metabolism and ME in physically active, age-, gender-, physical activity level-, and performance-matched participants with similar training backgrounds in order to standardize the exercise performance and the possible effects of earlier physical training. In this study, ME was 34% higher in the EF group and further, there was a negative correlation between $\dot{V}$O_2_ at 45% of $\dot{V}$O_2_peak and ME in the entire study population. The dispersion in ME within the entire study population was large, ranging from 14 to 27% but, however, the observed values are within the normal limits reported earlier (de Koning et al., 2012).

The main finding in the present study revealed significantly higher muscle FAU in the EF than in IE group and that FAU also correlated with ME. Muscle FAU depends on exercise intensity (Romijn et al., 1993), level of substrate delivery (Saltin et al., 1983), and substrate transport capacity from blood to the working muscle cells (Holloway et al., 2008; Sahlin and Harris, 2008). In the current study, FAU delivery was similar in the both groups, but FAU extraction was higher in the EF than in IE group indicating that the mechanisms associated with FAU transport from blood to muscle work better in the EF participants. This could be related to increased content of FAU transport protein (FAT/CD36) that has been proposed to act at both the muscle and mitochondrial membranes, thereby, increasing FAU transport into the muscle cells and mitochondrias (Smith et al., 2012). Exercise training has been shown to increase FAT/CD36 content (Talanian et al., 2010), which may therefore also be linked to ME. In addition, it has been earlier shown that ME during cycling associates to percentage of type I muscle fibers (Coyle et al., 1992; Horowitz et al., 1994) and percentage of MHC I (Mogensen et al., 2006). Thus, theoretically, the mitochondrial density and enzyme activities may play an important role in this regard. Indeed, higher oxidative enzyme activity has been linked to improved ME (Goldsmith et al., 2010). However, FAT/CD36 content has been shown to be important for increased fat oxidation independent on mitochondrial content and enzymes (Yoshida et al., 2013). Unfortunately, muscle biopsy samples were not taken in the present study and, therefore, cannot confirm the potential mechanism that needs to be studied in future experiments.

The increased FAU in the EF group could also be explained by changes in hormonal regulation. One of the hormonal candidates is insulin, which inhibits FAU transport across the plasma membrane (Spriet, 2014). Indeed, it has been shown that improved ME by high intensity interval training is linked to decreased fasting insulin concentration (Jabbour and Iancu, 2015). Thus, in the current study, insulin concentration was higher in the IE than in EF group, therefore fitting nicely to observed lower FAU in the IE group. However, analysis of glucagon concentration would have given additional information in this regard. Epinephrine and norepinephrine concentrations were also analyzed, but they were similar in the both groups and, therefore, cannot explain the differences in FAU between the groups.

The second hypothesis was that glucose uptake would be decreased in the EF compared to IE participants. However, the current study failed to demonstrate this and also glucose extraction and glucose delivery were similar between the groups. Thus, it seems that glucose metabolism does not play any significant role in determining differences in ME, at least at the exercise intensity used in the present study.

EE_tot_ was calculated based on the measured m$\dot{V}$O_2_. EE_tot_ in the entire study population during exercise corresponded approximately to the level of 50% of $\dot{V}$O_2_max during whole body exercise (Romijn et al., 2000). EE_tot_ was also similar in the both groups due to similar m$\dot{V}$O_2_ and RQ levels. Based on EE_tot_ and measured FAU and GU, assuming that these substrates were fully oxidized during exercise, also the use of intramuscular fats and carbohydrates was calculated. These calculations showed that only the use of plasma FAU was significantly different between the groups, while usage of blood glucose and intramuscular energy sources (intramuscular fat and glycogen) did not differ. In the entire study population, the usage of plasma FAU and glucose averaged to ≈14 and ≈5% of EE_tot_, respectively. These values are lower than earlier reported by Romijn et al. (2000) who suggested that usage of plasma FAU and glucose at the intensity of 50 % of $\dot{V}$O_2_max corresponds to ≈40 and ≈10%, respectively. However, methodological differences may explain this difference as in the present study FAU and GU were measured directly from working muscle, whereas in the study of Romijn et al. (2000), muscle substrate uptakes were determined at the whole body level. On the other hand, the use of intramuscular energy sources was not directly measured in the present study and several assumptions were made when calculating them. This may have led to overestimation of the use of intramuscular energy sources.

As muscle RQ levels were similar between groups, the total calculated fat and carbohydrate oxidation was similar in both groups. However, the use of plasma FAU was higher in EF groups. Thus, the higher FAU in EF group is likely due to enhanced FAU transport capacity between capillaries and muscle cells. This suggestion needs to be clarified in further studies, especially in endurance athletes, as our study participants were recreationally active healthy males without any regular participation to endurance training.

### Methodological Considerations and Study Limitations

This study was performed in relatively young untrained (average leisure-time physical activity 3.8 sessions per week) men and the results cannot therefore be directly extrapolated to other age groups, female participants or to participants with endurance exercise training background.

Positron emission tomography methodology was applied to investigate FAU and GU directly from the muscle tissue avoiding potential confounding effects of other tissues. On the other hand, the use of intramuscular energy sources was not directly measured and, therefore, those results should be cautiously interpreted. Also, the use of PET limited the exercise model that could be used. During PET scanning the motion should be minimized and consequently, cycling exercise during PET scanning is not possible. Therefore, completely different exercise models were used for the determination of ME (cycling) and muscle metabolism (one-leg knee-extension exercise). However, during both cycling and knee-extension exercise mainly concentric muscle actions are used. In addition, PET method provides an unique option to measure muscle metabolism non-invasively *in vivo*, which is a strength in this study. Also the use of a simple one-leg exercise model, where concentric muscle actions were emphasized, can also be considered as strength, as muscle intrinsic factors could be better investigated with this isolated model. However, due to invasive nature of the measurements, the sample size was rather low and therefore, larger sample size would have been needed, e.g., to get higher power for the present findings.

## Conclusion

The novel new finding of the present study was that muscle free fatty-acid uptake associated to ME during exercise. More efficient untrained, but physically active young men had increased muscle FAU compared to inefficient participants during one-legged knee-extension exercise which led to higher usage of plasma FAU in energy metabolism, while the use of blood glucose and intramuscular energy sources appeared to be similar between efficient and inefficient participants. These findings suggest that the effective use of plasma FAU is an important metabolic determinant of ME during exercise.

## Author Contributions

ML, HK, JKn, and KK designed the study. ML, JKe, and KK collected the data. ML and KK analyzed the data. ML, HK, JKe, JKn, and KK interpreted the results. ML, HK, and KK wrote the draft. JKe and JKn revised the manuscript. ML, HK, JKe, JKn, and KK approved the final version to be published and agreed to be accountable for all aspects of the work.

## Conflict of Interest Statement

The authors declare that the research was conducted in the absence of any commercial or financial relationships that could be construed as a potential conflict of interest.
